# Management Strategies for Seminal Vesicle Cysts in Zinner Syndrome: Insights From Two Cases

**DOI:** 10.7759/cureus.87348

**Published:** 2025-07-05

**Authors:** Fadhel Yusuf, Abdulla Husain, Zuhair Sikafi, Salam Alhasani, Mudhar N Hasan

**Affiliations:** 1 Urology, Dubai Hospital, Dubai, ARE; 2 Radiology, Dubai Health, Dubai, ARE; 3 Urology, Mediclinic City Hospital, Dubai, ARE; 4 Urology, Mediclinic Welcare Hospital, Dubai, ARE

**Keywords:** ejaculatory duct obstruction, renal agenesis, robotic surgery, seminal vesicle cyst, zinner syndrome

## Abstract

Zinner syndrome is a rare congenital malformation that affects the male genitourinary tract, characterized by a triad of unilateral renal agenesis, seminal vesicle cysts, and ejaculatory duct obstruction, and typically presents during the third or fourth decade of life. In this report, we present two cases illustrating distinct management pathways. One patient, with mild and stable symptoms, was successfully managed with conservative observation. The second patient, following failed medical therapy, underwent a successful laparoscopic robotic-assisted vesiculectomy with significant symptom improvement. These cases highlight the clinical variability associated with Zinner syndrome and reinforce the importance of a tailored, patient-specific approach to management. They also contribute to the growing literature supporting minimally invasive surgical techniques as a safe and effective option for selected symptomatic patients with Zinner syndrome, offering favorable outcomes with reduced morbidity.

## Introduction

Zinner syndrome, first described by Zinner in 1914, is a rare congenital anomaly of the Wolffian duct, affecting the genitourinary system in male patients [1]. It is often characterized by a triad of unilateral renal agenesis, seminal vesicle cysts, and ejaculatory duct obstruction, and usually manifests during the third or fourth decade of life [2,3]. The embryological origin of Zinner syndrome involves abnormal development of the mesonephric duct during early fetal life [4]. The majority of patients remain asymptomatic until adulthood, when they may present with a variety of symptoms, including perineal pain, dysuria, painful ejaculation, and infertility [5]. The absence of the ipsilateral kidney is a hallmark finding in Zinner syndrome, often leading to compensatory hypertrophy of the contralateral kidney [6]. Diagnosis is typically made through imaging, with ultrasound and MRI providing detailed visualization of seminal vesicle cysts and associated abnormalities [7]. Early recognition of the syndrome is essential for optimal management, particularly in preventing complications such as testicular atrophy and chronic pain [8].

For asymptomatic patients diagnosed incidentally, surveillance is often the preferred management option [6]. In contrast, symptomatic patients may require intervention or surgical procedures if they do not respond to initial conservative medical therapy [9]. Treatment options for Zinner syndrome include antibiotic therapy for infected, thickened collections and urinary tract infections (UTIs), percutaneous image-guided aspiration, sclerosant injection, transurethral resection of the ejaculatory ducts, or surgery. Surgical approaches can be open, laparoscopic, or, more recently, robot-assisted laparoscopic surgery has also been reported for Zinner syndrome [10]. Currently, there is no established preferred treatment method due to the limited literature and the small number of reported cases.

## Case presentation

Case 1

**Figure 1:**
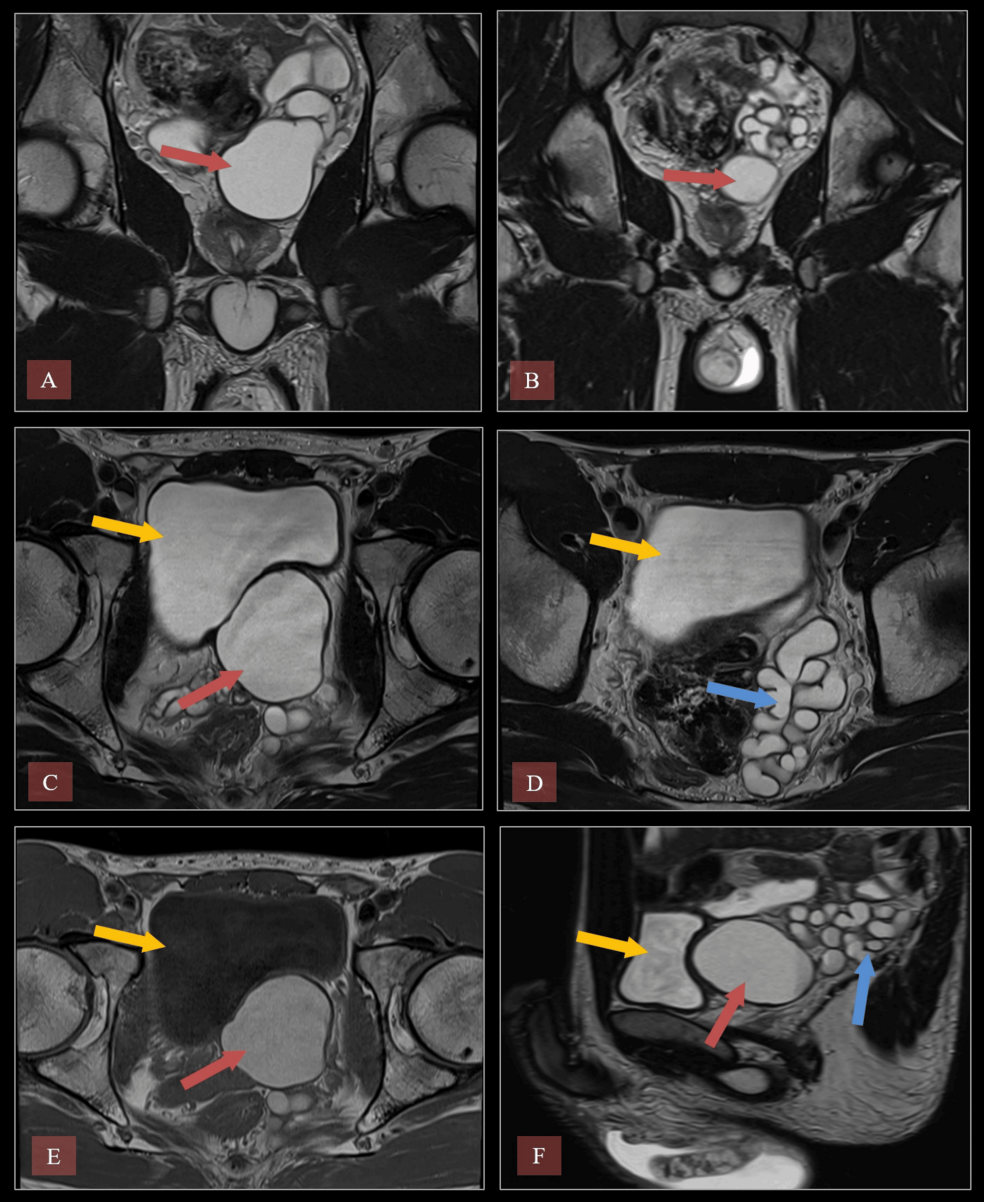
MRI of the pelvis demonstrating a left seminal vesicle cyst and dilated seminal vesicle. (A) Coronal T2-weighted MRI. The red arrow indicates the seminal vesicle cyst. (B) Coronal T2 TSE. The red arrow indicates the seminal vesicle cyst. (C) Axial T2 TSE. The yellow arrow indicates the bladder, and the red arrow indicates the seminal vesicle cyst. (D) Axial T2 TSE. The yellow arrow indicates the bladder, and the blue arrow indicates the dilated seminal vesicle. (E) Axial T1 TSE. The yellow arrow indicates the bladder, and the red arrow indicates the seminal vesicle cyst. (F) Sagittal T2 TSE. The yellow arrow indicates the bladder, the red arrow indicates the seminal vesicle cyst, and the blue arrow indicates the dilated seminal vesicle. TSE: turbo spin echo

A 33-year-old man presented with left testicular pain, difficulty urinating, and a palpable left testicular lump, which he had first noticed three months earlier. Scrotal ultrasound revealed a hypoechoic area in the left testis with increased vascularity and absence of the left kidney. MRI confirmed left seminal vesicle cystic dilation, left vas deferens dilatation, and left ejaculatory duct obstruction (Figure 1), suggesting Zinner syndrome, along with left testicular atrophy.

The prostate was normal in size on imaging. Despite stable, unchanged symptoms, the patient was monitored with repeat MRI, which demonstrated no significant interval changes. Conservative management with regular follow-up was initiated, including tamsulosin 0.4 mg nightly, which led to symptomatic improvement and supported the decision to continue non-surgical management. The patient remained asymptomatic upon routine clinical follow-up, was fully aware of his diagnosis and its potential implications, and continues under observation without the need for intervention.

Case 2

A 32-year-old man presented with a five-year history of lower urinary tract symptoms (LUTS), with frequent urination and nocturia being the most pronounced symptoms. An initial evaluation was conducted with ultrasound of the kidneys, ureters, and bladder (KUB) and pelvic MRI to investigate the underlying cause of his symptoms. An abdominal and pelvic ultrasound revealed key findings, including left renal agenesis. The right kidney appeared normal, with no evidence of renal stones or hydronephrosis. The ultrasound also identified a large trabeculated cyst at the left side of the urinary bladder base, which raised concerns for further structural abnormalities.

**Figure 2:**
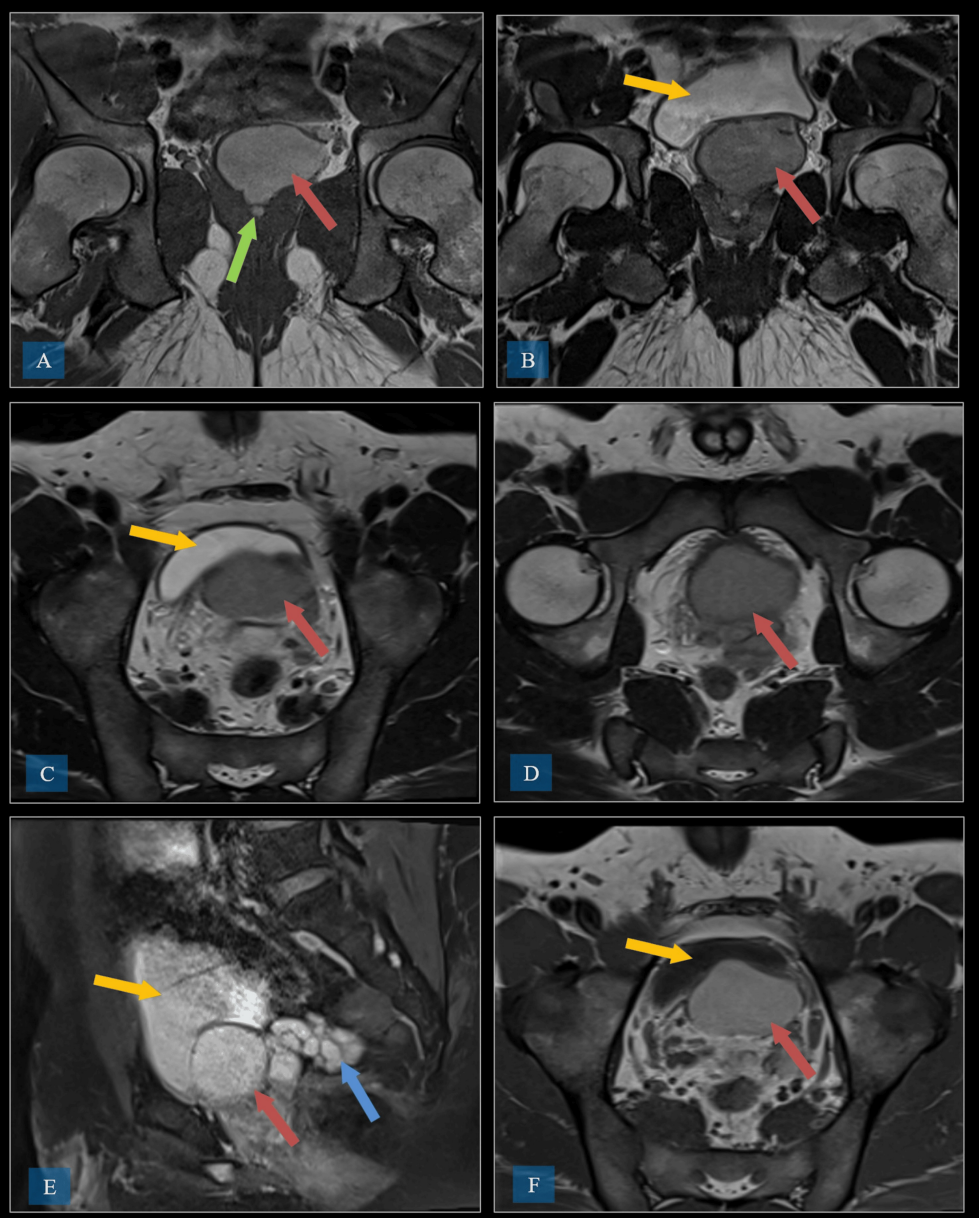
MRI of the pelvis demonstrating a large convoluted cystic dilatation of the left seminal vesicle measuring approximately 6 cm in diameter. (A) Coronal T1-weighted MRI. The red arrow indicates the left seminal vesicle cyst, and the green arrow indicates the ejaculatory duct obstruction. (B) Coronal T1-weighted MRI. The red arrow indicates the left seminal vesicle cyst, and the yellow arrow indicates the urinary bladder. (C) Axial T2 TSE. The red arrow indicates the left seminal vesicle cyst, and the yellow arrow indicates the urinary bladder. (D) Axial T2 TSE. The red arrow indicates the left seminal vesicle cyst. (E) Sagittal T1 TSE. The red arrow indicates the left seminal vesicle cyst, the yellow arrow indicates the urinary bladder, and the blue arrow indicates the dilated left seminal vesicle. (F) Axial T1 TSE. The red arrow indicates the left seminal vesicle cyst, and the yellow arrow indicates the urinary bladder. TSE: turbo spin echo

MRI of the pelvis was performed, which revealed a large convoluted cystic dilatation of the left seminal vesicle measuring approximately 6 cm in diameter (Figure 2).

The cyst was filled with high-proteinaceous fluid, as indicated by T2 hypointense and T1 bright signals, and extended down to the level of the ejaculatory ducts. This caused funneling through the transitional lobe of the prostate, displacing both peripheral lobes. The appearance was suggestive of an ejaculatory duct stricture or obstruction, but there was no post-contrast enhancement or communication with the urinary bladder or distal left ureter.

**Figure 3:**
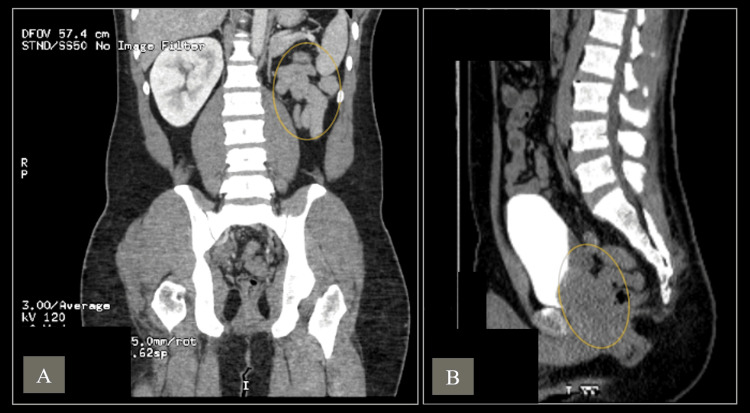
Contrast-enhanced CT of the abdomen and pelvis demonstrating congenital left renal agenesis and a large seminal vesicle cyst measuring 7 × 5 cm. (A) Coronal view. The yellow circle highlights the congenital absence of the left kidney (renal agenesis). (B) Sagittal view. The yellow circle highlights the 7 × 5 cm left seminal vesicle cyst.

A CT scan with contrast of the abdomen further characterized the seminal vesicle cyst, showing approximately 7 x 5 cm seminal vesicle cyst (Figure 3).

It was displacing and indenting the posterior and inferior walls of the urinary bladder, particularly at the trigonal region, but without causing significant urinary obstruction or backpressure. There was also no communication with the bladder or ureters. Additionally, the CT scan revealed the absence of the left kidney, confirming a diagnosis of congenital left renal agenesis, which was seen on abdominal ultrasound initially. This was associated with compensatory hypertrophy of the right kidney, which measured 14 cm in its bipolar diameter.

Given the combination of left renal agenesis, seminal vesicle cyst, and ejaculatory duct obstruction, the patient was diagnosed with Zinner syndrome.

Initially, conservative management was attempted with the use of alpha-blockers (alfuzosin) for four weeks to alleviate the LUTS. However, upon follow-up, the patient reported no significant improvement in his symptoms. Therefore, after careful consideration of treatment options, including aspiration versus surgical excision, the decision was made to proceed with robotic-assisted vesiculectomy, as the large cyst was causing persistent symptoms and impacting the patient's quality of life.

**Figure 4:**
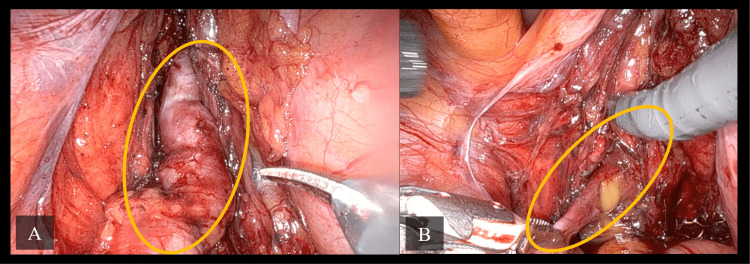
Intraoperative images from robotic-assisted vesiculectomy demonstrating the left seminal vesicle cyst. (A) Intraoperative view shows the left seminal vesicle cyst (yellow circle) prior to excision. (B) Intraoperative view shows fluid expression from the left seminal vesicle cyst (yellow circle) during incision, which confirms its cystic nature.

Approximately one month later, the patient underwent a robotic-assisted vesiculectomy. During the procedure, infected fluid was drained from the cyst (Figure 4). The seminal vesicle cyst was successfully removed, and a catheter was placed at the end of the procedure.

Postoperatively, the patient followed an appropriate recovery trajectory, with clear urine in the catheter, good mobilization, and was managed on oral analgesia and antibiotics. He was reassessed the following day before discharge with a plan for catheter removal 10 days after the surgery date. When he returned to the clinic 10 days later, both the catheter and sutures were successfully removed. He reported no LUTS and no surgical incision complications. Histopathology later revealed a benign seminal vesicle cyst.

## Discussion

Zinner syndrome is a rare congenital malformation, often considered the male counterpart of Mayer-Rokitansky-Küster-Hauser syndrome in female patients [11]. The condition arises due to defective embryogenesis of the mesonephric (Wolffian) duct during the first trimester of pregnancy, which affects the development of the kidneys, seminal vesicles, and ejaculatory ducts [12]. Individuals are generally diagnosed in their 30s or 40s and often exhibit symptoms such as pain in the perineal area, recurrent prostatitis, blood in the semen, discomfort during ejaculation, and issues with infertility [13]. Regardless of the imaging method, the key findings include the classic triad of unilateral renal agenesis, ipsilateral obstruction of the ejaculatory duct, and ipsilateral seminal vesicle cyst [13].

The first case, a 33-year-old male patient, exhibited the typical triad of Zinner syndrome, including the absence of the left kidney, cystic dilatation of the left seminal vesicle, and ejaculatory duct obstruction. Initial ultrasound revealed a hypoechoic area in the left testis, alongside increased vascularity. The absence of the left kidney, as confirmed by imaging, is a classic feature of Zinner syndrome [14]. Further investigation through MRI revealed significant dilation of the left vas deferens and seminal vesicles, consistent with previously reported cases of the syndrome [15]. The atrophy of the left testis, with patchy areas of T2 hypointensity, suggested secondary damage due to the chronic obstruction and dilation of the seminal vesicles [16]. Zinner syndrome patients often present with complaints of perineal pain, dysuria, or painful ejaculation due to the obstruction of seminal fluid drainage [17].

In the first case, the patient's symptoms included left testicular pain and difficulty in urination, both common clinical features. While many patients with Zinner syndrome remain asymptomatic until adulthood, the increasing pressure on the ejaculatory ducts and seminal vesicles due to cystic dilation can lead to significant discomfort [6]. The role of imaging is critical in diagnosing Zinner syndrome. Ultrasound typically reveals seminal vesicle cysts, while MRI provides superior soft tissue contrast, allowing for a more detailed assessment of the seminal vesicles, ejaculatory ducts, and surrounding structures [8]. In this case, MRI findings of gross dilatation in the vas deferens and seminal vesicles, as well as thickening of the left spermatic cord, were key to the diagnosis. Follow-up MRI showed no significant changes, indicating that the condition was stable. Management of Zinner syndrome is individualized and depends on the severity of symptoms. In asymptomatic patients, conservative management with regular follow-up is often sufficient [6].

The second case, a 32-year-old male patient with a five-year history of LUTS, was diagnosed with Zinner syndrome, characterized by left renal agenesis, a large seminal vesicle cyst, and ejaculatory duct obstruction. Imaging, including MRI and CT, revealed a 7 x 5 cm seminal vesicle cyst displacing the bladder wall without causing significant obstruction. Initial conservative management with alpha-blockers failed to improve symptoms, leading to a robotic-assisted vesiculectomy. The cyst was successfully removed, and the patient recovered well postoperatively. In conclusion, for patients experiencing significant symptoms who fail conservative medical therapy, surgical options like cyst aspiration, transurethral resection of the ejaculatory ducts, or surgical cyst removal may be required [6]. Surgical approaches for Zinner syndrome can include open surgery, laparoscopic techniques, and, more recently, robot-assisted laparoscopic surgery [10]. 

Although Zinner syndrome includes obstruction of the ejaculatory duct, fertility may be preserved in many cases due to the typically unilateral nature of the condition, with the opposite ejaculatory duct and seminal vesicle remaining functional. In our case series, both patients demonstrated preserved fertility despite the presence of seminal vesicle cysts and unilateral obstruction. Nonetheless, infertility remains a potential concern, as ejaculatory duct obstruction has been reported in up to 45% of boys with Zinner syndrome [18]. Since evaluating fertility during adolescence is limited, it is advisable that these patients are enrolled in structured follow-up programs, with semen analysis recommended once they reach adulthood to assess reproductive potential. The current literature offers limited discussion on long-term fertility outcomes in this population, highlighting the need for further research.

## Conclusions

Zinner syndrome is a rare but clinically important congenital condition involving developmental anomalies of the mesonephric duct. While some patients remain asymptomatic, others may present with LUTS, pelvic or ejaculatory pain, or concerns related to fertility. Early diagnosis, ideally through MRI of the pelvis, along with a careful evaluation of the clinical presentation, is essential for guiding appropriate management. 

Treatment approaches should be individualized, ranging from conservative follow-up in stable or asymptomatic cases to surgical intervention in those with persistent or bothersome symptoms. Surgical excision of the seminal vesicle cyst remains the standard for symptomatic patients. The evolution of minimally invasive and robotic-assisted surgical techniques has further improved treatment outcomes by reducing morbidity and enhancing recovery.
